# SLCO4C1 promoter methylation is a potential biomarker for prognosis associated with biochemical recurrence-free survival after radical prostatectomy

**DOI:** 10.1186/s13148-019-0693-2

**Published:** 2019-07-09

**Authors:** Xin Li, Wanfeng Zhang, Jing Song, Xianqin Zhang, Longke Ran, Yunfeng He

**Affiliations:** 1 Department of Urology, The First Affiliated Hospital of Chongqing Medical University, Chongqing, 400016 China; 2 Department of Bioinformatics, The Basic Medical School of Chongqing Medical University, Chongqing, 400016 China; 3 Molecular Medicine and Cancer Research Center, Chongqing Medical University, Chongqing, 400016 China

**Keywords:** Prostate cancer, DNA methylation, Prognosis, Biomarker

## Abstract

**Background:**

Prostate cancer (PC) is a commonly diagnosed malignancy in males, especially in the western hemisphere. The extensive use of multiple biomarkers plays an important role in the diagnosis and prognosis of PC. However, the accuracy of biomarkers for PC prognosis needs to be urgently improved. This study aimed to identify a novel prognostic biomarker for PC.

**Materials and methods:**

Differentially methylated CpG sites were identified from the GSE76938 dataset (https://www.ncbi.nlm.nih.gov/geo/) using R software version 3.1.4. Four significant CpG sites on the SLCO4C1 gene were found to be closely associated with prognosis in PC. Data downloaded from The Cancer Genome Atlas (TCGA) were used for validation. Co-expression and functional enrichment analyses were used to explore the roles of SLCO4C1 in molecular functions, biological processes and cellular components. Total RNA extraction and qRT-PCR were used to reveal the difference in SLCO4C1 expression between tumour and normal tissues. Bisulfite amplicon sequencing (BSAS) was used to identify methylation levels at the CpG sites.

**Results:**

In the GSE76938 cohort, 10,206 CpG sites were identified to be differentially methylated in tumour versus normal prostate tissues. Among the CpG sites, four sites (cg06480736, cg19774478, cg19788741 and cg22149516) located in the promotor region (TSS200-1500) of SLCO4C1 were found to be significantly hypermethylated in tumour tissues. The results were validated in an independent dataset (TCGA PRAD cohort). In the cohort from TCGA, SLCO4C1 expression was negatively correlated with methylation levels at the four sites. The results of qRT-PCR validated that tumour tissues had a relatively lower expression of SLCO4C1. Bisulfite amplicon sequencing (BSAS) further confirmed a higher methylation level at the SLCO4C1 promoter in tumour tissues. SLCO4C1 (cg06480736, cg19774478, cg19788741 and cg22149516) was identified as a significant promising biomarker for biochemical recurrence-free survival in Kaplan-Meier analysis (*P* < 0.01) and univariate Cox proportional hazards analysis: cg06480736 (HR 15.914, *P* < 0.001), cg19774478 (HR 9.001, *P* < 0.001), cg19788741 (HR 10.759, *P* = 0.003) and cg22149516 (HR 17.144, *P* = 0.006). However, three sites, namely, cg06480736 (HR 1.809, *P* = 0.049), cg19774478 (HR 1.903, *P* = 0.041) and cg22149516 (HR 2.316, *P* = 0.008), were confirmed in multivariate analysis.

**Conclusions:**

SLCO4C1 promoter methylation, including that at three CpG sites, namely, cg06480736, cg19774478 and cg22149516, is a potential biomarker for risk stratification and might offer significantly relevant prognostic information for PC patients after radical prostatectomy.

## Background

Prostate cancer (PC) is a common malignancy in males, especially in the western hemisphere. PC accounts for 9.9% of all new tumors [1]. This disease has become a serious threat to the health of elderly men. A recent study revealed 164,690 new PC patients and 29,430 deaths in the USA in 2018 [2]. As a biomarker of prostate cancer, PSA (prostate-specific antigen) plays an important role in early diagnosis. Most PC cases can be diagnosed early when curative treatment is still possible. However, excess use of PSA testing often leads to overdiagnosis and overtreatment, increasing the economic burden and reducing the quality of life for men [3]. Radical prostatectomy is currently the main curative treatment for prostate cancer. Nevertheless, more than 30% of prostate cancer patients experience biochemical recurrence (BCR) after radical prostatectomy within 10 years, and many of these patients may develop metastatic castration-resistant PC (CRPC) [4].

Prostate cancer can be divided into aggressive and indolent subtypes. Commonly, PC in most patients progresses slowly [4]. However, many PC cases are rapidly progressing and may develop metastasis within a short period of time. This is one of the main factors leading to the poor prognosis of these patients. The distinction between aggressive and indolent prostate cancer is very important and remains a challenge for clinical management. Currently, PC prognosis is mainly based on clinicopathological parameters such as PSA level, clinical stage and Gleason score. Nevertheless, these tools are still not adequate for early diagnosis. Therefore, more accurate and improved prognostic biomarkers for PC are urgently needed.

The epigenetic mechanism of DNA methylation plays an important role in the biological behaviour of human malignancies. DNA methylation, especially aberrant promoter methylation, is often found to be involved in tumour cell proliferation and differentiation [5]. Since DNA methylation can be quantified in tumour and normal tissues, methylation-based biomarkers have been proven to be diagnostic and prognostic tools for human malignancies [6–8]. Many DNA methylation biomarkers, such as PITX2, PD-L1 and GSTP1, have been proven to be efficient biomarkers in the prognosis of PC [9, 10]. The aim of this study was to identify and evaluate potential biomarkers for prognosis based on biochemical recurrence-free survival after radical prostatectomy for PC.

## Results

### SLCO4C1 promoter had higher methylation levels in PC tissues and the gene transcription was decreased from the GEO database

**Fig. 1:**
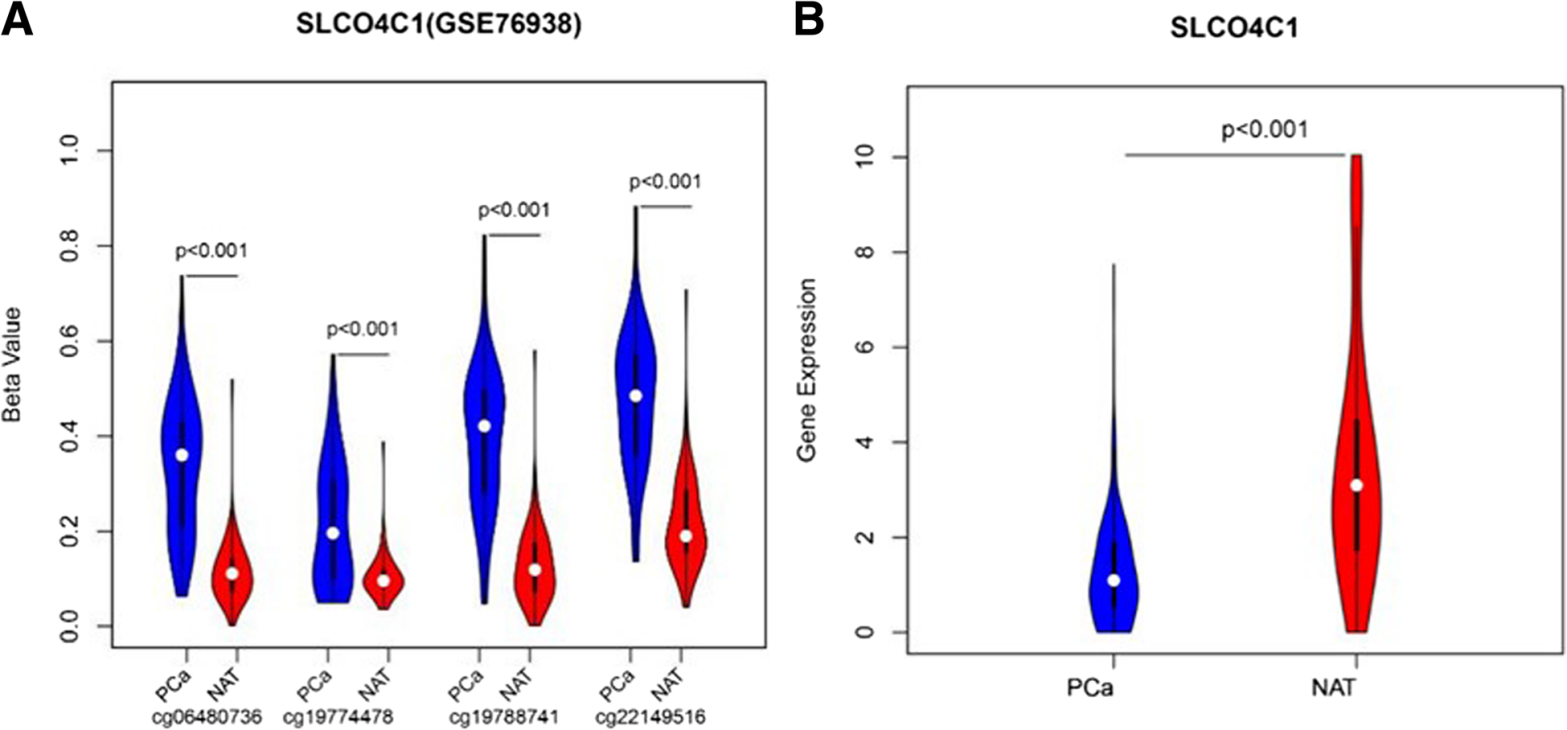
SLCO4C1 methylation and mRNA expression level in GSE76938 dataset. **a** Four CpG sites methylation level (beta value) of SLCO4C1 in tumour (PC) and normal tissue. **b** The differential gene expression in tumor and normal tissue

For the exploration of significant methylation sites in prostate cancer tissues, the GSE76938 dataset, including 73 primary PC and 63 adjacent samples downloaded from the Gene Expression Omnibus (GEO), was analysed with the limma package in R software 3.1.4. These samples were profiled using the Illumina Human Methylation 450 platform, which includes 485,577 CpG sites. To explore differentially methylated CpG sites (*P* < 0.001, ∣Delta Beta∣ > 0.2) between PC and adjacent samples, these CpG sites were assessed by the limma package for linear regression model analysis. After removing the differentially methylated CpG sites that were not located in the promoter region (TSS200-1500), a total of 10,206 differentially methylated CpG sites in 2182 gene promoters were identified. Then, 59 candidate genes that were negatively correlated with gene expression were found. In particular, four differentially methylated CpG sites (cg06480736, cg19774478, cg19788741 and cg22149516) were located in the promoter region of SLCO4C1. Then, we have checked that there was no significant difference in the number of probes of these 59 candidate genes. Based on further analysis of the data (GSE76938), the four sites displayed significantly higher levels of methylation in PC tissues than in normal tissue (Fig. 1a), while the transcription of SLCO4C1 was decreased (*P* < 0.001, Fig. 1b).

### SLCO4C1 promoter differential methylation and mRNA expression was validated in TCGA database

**Fig. 2:**
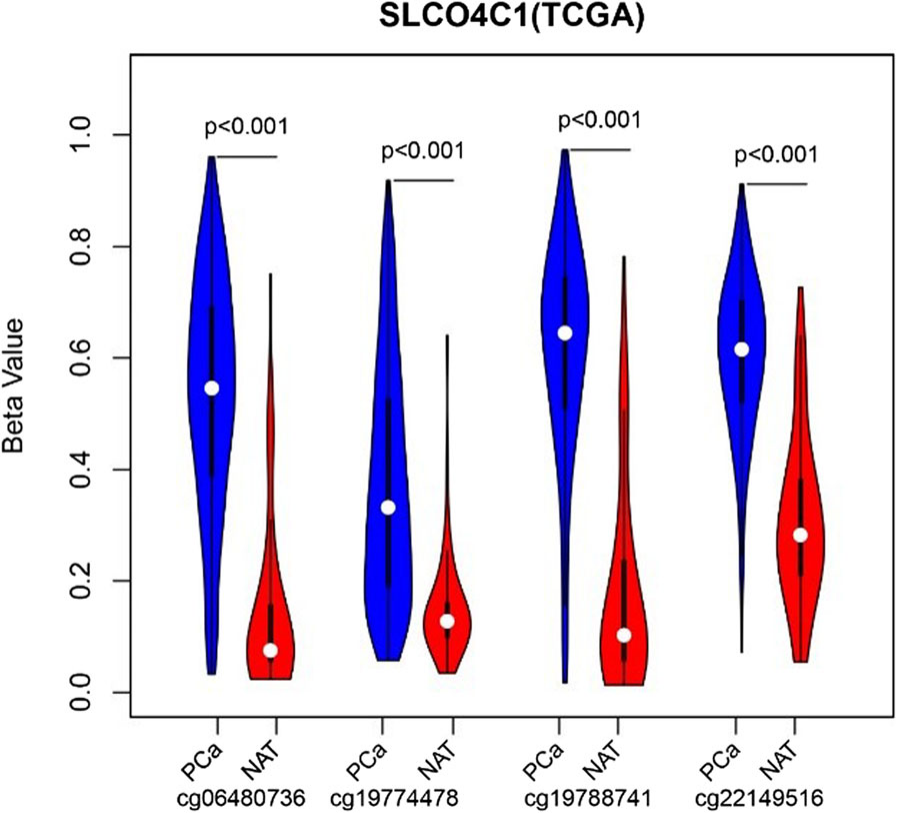
Four CpG sites (cg06480736, cg19774478, cg19788741, cg22149516) methylation level (Beta value) of SLCO4C1 in tumor (PC) and normal tissue (NAT)

To validate differential methylation at the four CpG sites (cg06480736, cg19774478, cg19788741 and cg22149516) of SLCO4C1, we also analysed a validation cohort of 498 primary PC and 50 adjacent samples from TCGA database with the limma package in R software 3.1.4. These samples were also profiled using the Illumina Human Methylation 450 platform. Finally, these four CpG sites of SLCO4C1 were validated, showing significantly different methylation levels between adjacent tissues and tumour tissues in TCGA cohort. SLCO4C1 in tumour tissues showed significantly higher levels of promoter methylation than that in normal tissues (Fig. 2).

**Fig. 3:**
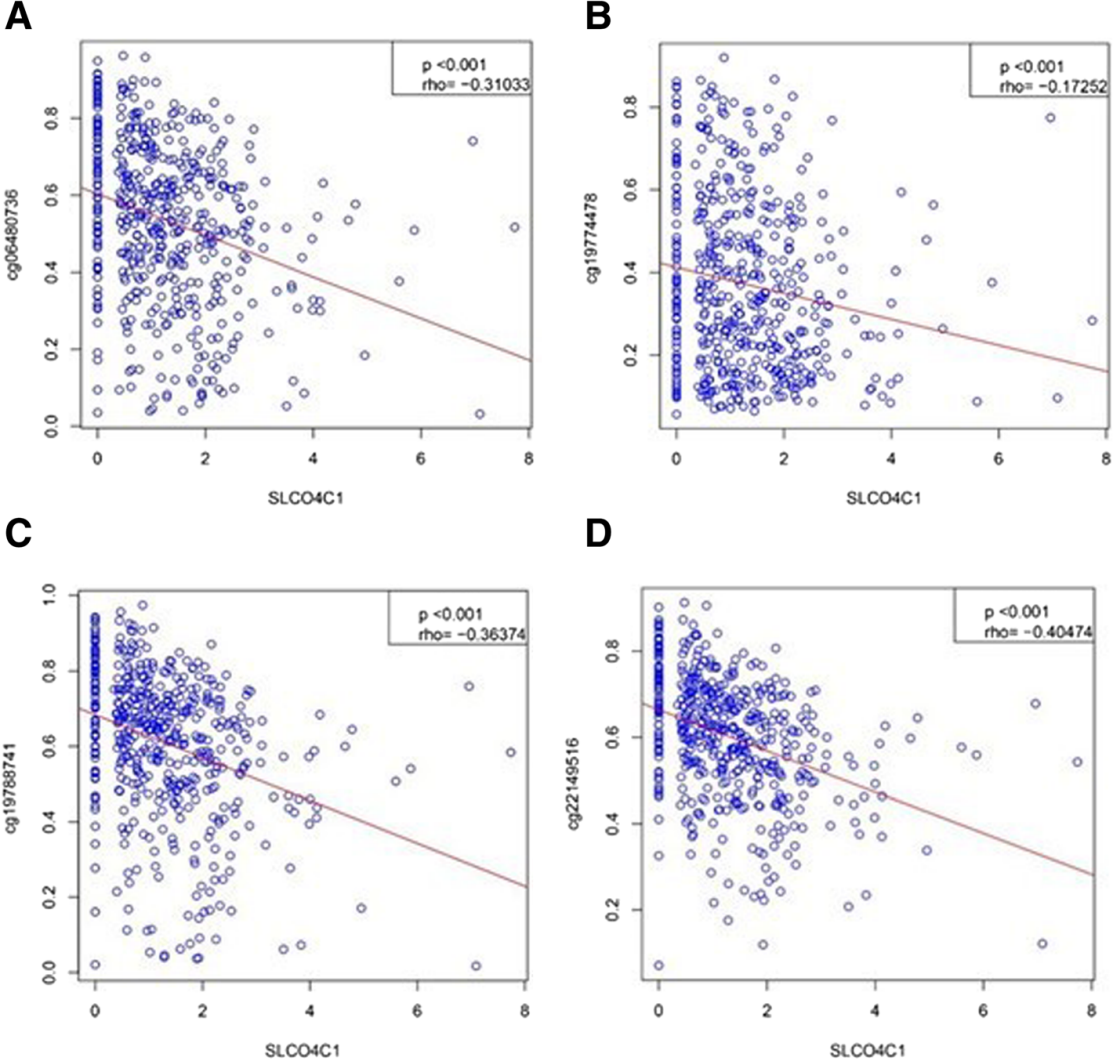
Association of four CpG sites methylation and SLCO4C1 mRNA expression in TCGA. **a** Association of cg06480736 methylation and mRNA expression. **b** Association of cg19774478 methylation and mRNA expression. **c** Association of cg19788741 methylation and mRNA expression. **d** Association of cg22149516 methylation and mRNA expression

To analyse the association of SLCO4C1 promoter methylation and mRNA expression, we used scatter plots to evaluate the correlation between four CpG sites and SLCO4C1 mRNA expression in TCGA database. SLCO4C1 expression was negatively correlated with the levels of methylation at these four CpG sites (*P* < 0.001, Fig. 3).

### SLCO4C1 significantly influenced biological processes

**Fig. 4:**
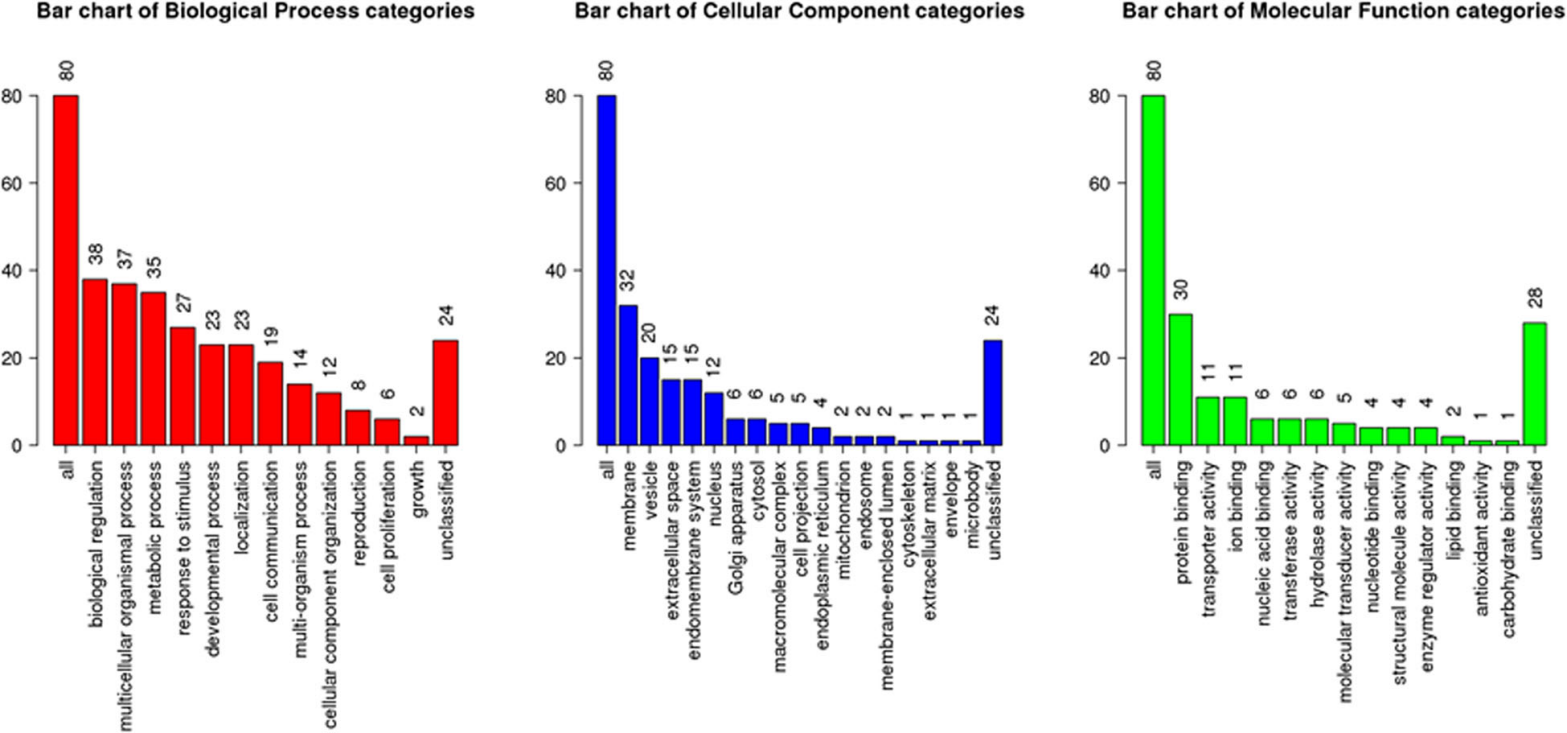
Functional enrichment analysis. The function of the top 100 co-expressed genesof SLCO4C1 (biological process, cellular component and molecular function)

The top 100 genes co-expressed with SLCO4C1 selected from PC profiles in TCGA database were included in WebGestalt (an online tool) to perform gene ontology analysis. The results showed that these genes were significantly enriched in biological regulation, cell proliferation and other biological processes, including molecular function with protein binding and transferase activity, cell projection and cytoskeleton cellular components (Fig. 4).

**Fig. 5:**
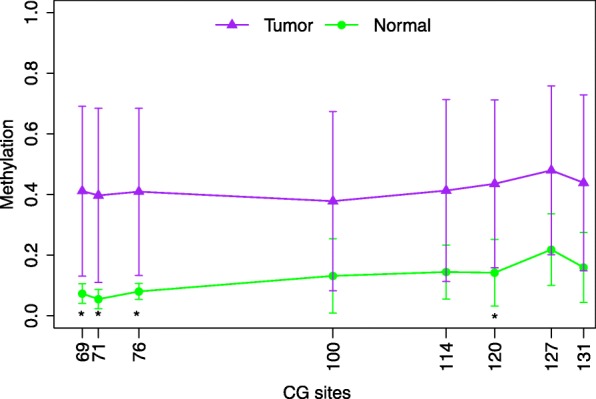
Schematic map of methylation information for all sites in tumor tissues and normal tissues. Asterisk represents Kruskall-Wallis P value < 0.05.

### Analytical assay design and bisulfite amplicon sequencing (BSAS) performance

**Fig. 6:**
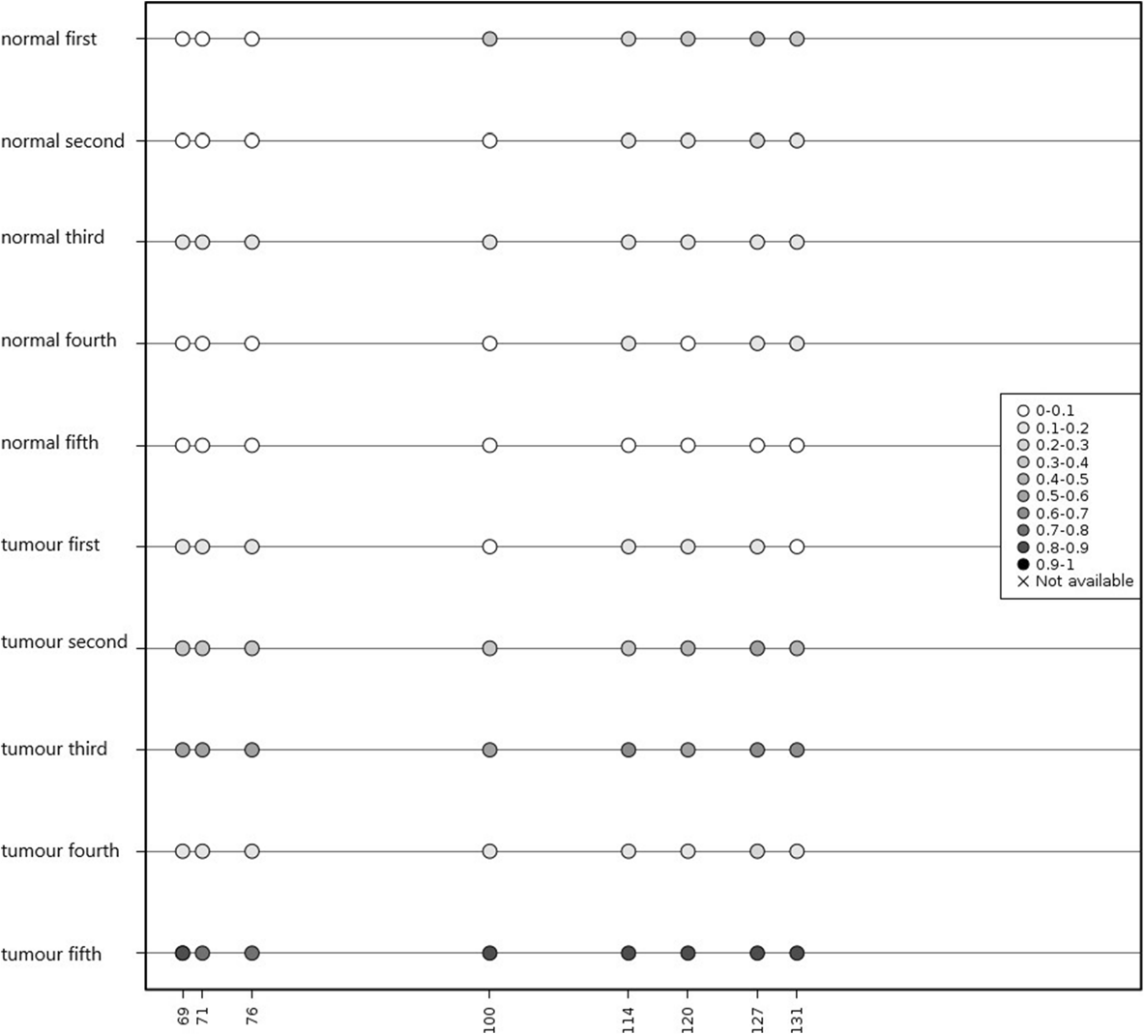
Schematic map of methylation information for all sites in tumor tissues and normal tissues. Asterisk represents Kruskall-Wallis *P* value < 0.05

To further analyse the methylation level at the SLCO4C1 promoter, we performed bisulfite amplicon sequencing (BSAS) on the CpG sites of the SLCO4C1 promoter. The results showed that four CpG sites (cg06480736, cg19774478, cg19788741 and cg22149516) were in the target regions with eight CG sites. The amplified fragment and CG sites were as follows: 5'-. Then, MethylKIT software (http://www.bioconductor.org/packages/release/bioc/html/methylKit.html) was used to analyse methylation data and calculate the average methylation level at all CpG sites. Further calculations showed that there was a significant difference in the average level of methylation at these CpG sites between tumour tissues and normal tissues (Kruskal-Wallis *P* value < 0.05). The CpG sites (cg06480736, cg19774478, cg19788741 and cg22149516) overlapped with points 69, 71, 76 and 120, respectively, in Fig. 6. Therefore, the previous analysis confirmed the presence of a high level of methylation at the SLCO4C1 promoter in tumour tissues and a relatively low level of methylation in normal tissues (Figs. 5 and 6).

**Fig. 7:**
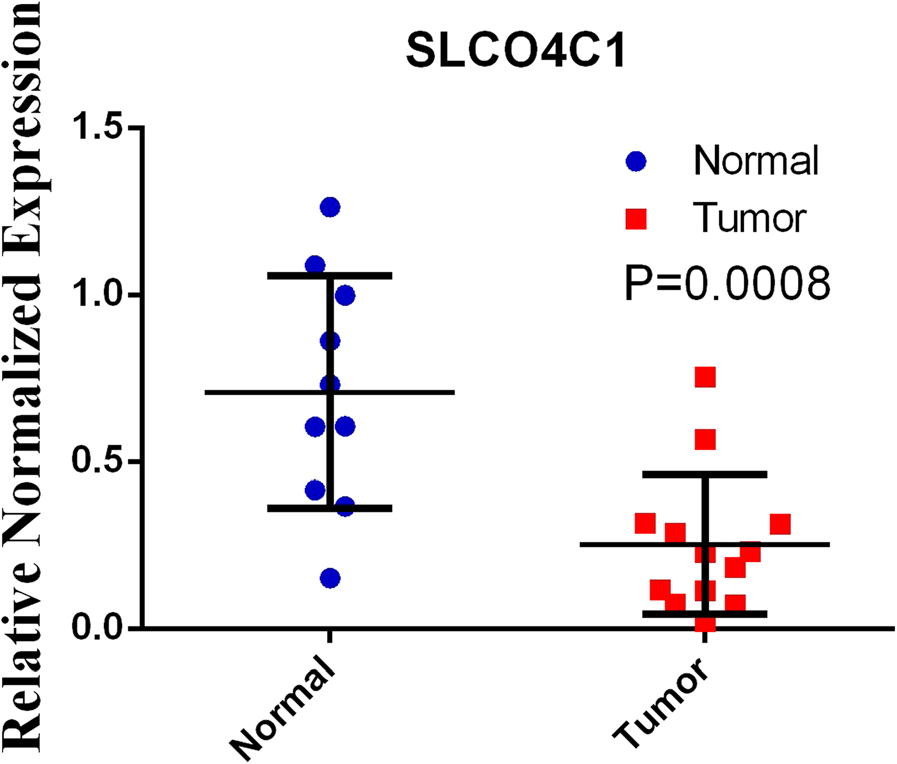
qRT-PCR results of SCLOC41 expression between normal and tumor tissues

### The expression of SLCO4C1 was negatively correlated with methylation levels

The difference in SLCO4C1 expression between tumour and normal tissues was quantified by qRT-PCR. After extracting total RNA using TRIzol reagent (TaKaRa), cDNA reverse transcription and qRT-PCR were performed. The qRT-PCR results revealed that SLCO4C1 was significantly downregulated in PC samples, indicating that the expression of SLCO4C1 was negatively correlated with methylation levels at these four sites (Fig. 7).

### Clinicopathologic correlation (TCGA cohort)

The Mann-Whitney *U* test and Kruskal-Wallis test were used to analyse the correlation between SLCO4C1 methylation and clinicopathological parameters (Table 1). The study showed that advanced tumour stages (pT) were associated with higher methylation levels at SLCO4C1 CpG sites (cg06480736, cg19774478, cg19788741 and cg22149516). Methylation levels at two sites (cg06480736 and cg19788741) were more likely to be associated with younger age. Except for that at the site cg22149516, the methylation level at of the other sites were related to Gleason score, surgical margin status and the nodal category. In contrast, preoperative PSA values were not associated with higher methylation levels at any of the four sites of the SLCO4C1 promoter. SLCO4C1 may play an important role in PC development and may be a novel biomarker for PC diagnosis and prognosis.

**Table 1:** Correlation between SLCO4C1 DNA promoter methylation and clinicopathological parameters

Variable	All patients	cg22149516 (*P* value)	cg06480736 (*P* value)	cg19788741 (*P* value)	cg19774478 (*P* value)
Patient number	498				
Age (years)					
> 60	247				
≤ 60	251	0.09^a^	0.04^a^	0.03^a^	0.36^a^
Tumour category					
pT2	188				
pT3 and pT4	303				
Unknown	7	0.02^a^	< 0.01^a^	< 0.01^a^	< 0.01^a^
Gleason score					
< 7	45				
3 + 4	147				
4 + 3	101				
= 8	64				
> 8	141	0.28^a^	< 0.01^a^	< 0.01^a^	< 0.01^a^
Surgical margin					
R0	316				
R1, R2 and RX	162				
Unknown	20	0.06^a^	< 0.01^a^	< 0.01^a^	0.02^a^
Nodal category					
pN0	346				
pN1	79				
Unknown	73	0.18^a^	0.02^a^	0.01^a^	0.01^a^
Preoperative PSA [ng/ml]					
0~4	51				
4–10	286				
> 10	153				
Unknown	8	0.64^b^	0.28^b^	0.42^b^	0.25^b^

^a^Mann-Whitney *U* test

^b^Kruskal-Wallis test

### Prognostic potential of SLCO4C1

**Fig. 8:**
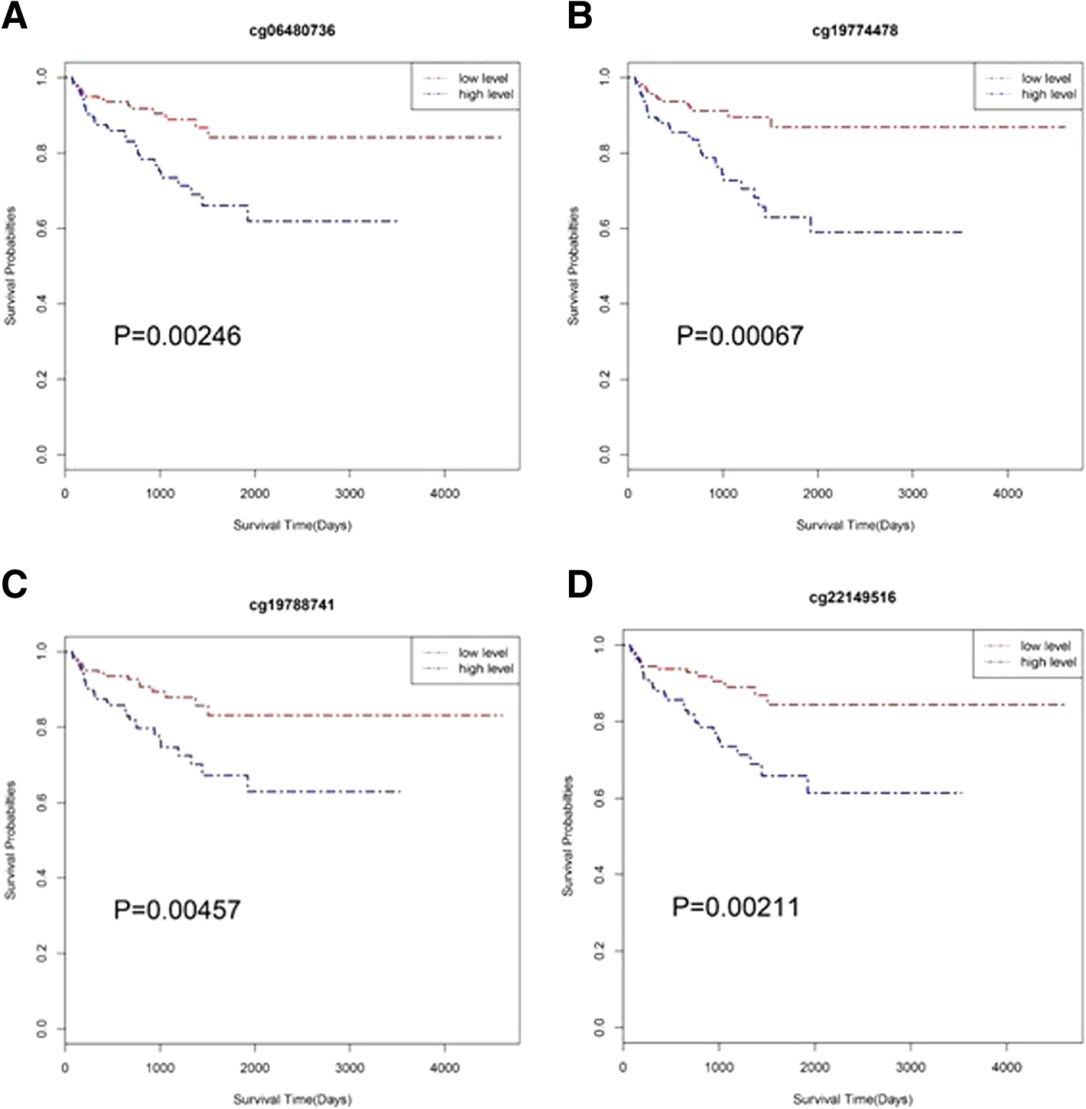
Kaplan-Meier survival analysis of BCR for the four sites: **a** cg06480736, **b** cg19774478, **c** cg19788741, **d** cg22149516. Red line indicates low methylation level. Blue line indicates high methylation level

To analyse the prognostic potential of SLCO4C1, we grouped 498 PC samples from TCGA according to low and high methylation levels by the median value. Kaplan-Meier analysis showed that all four sites of the SLCO4C1 promoter were significantly associated with BCR, and high methylation levels at the SLCO4C1 promoter predicted poor outcomes for PC (Fig. 8). Univariate analysis showed that BCR-associated factors were significantly related to T stage, grade, preoperative PSA values and methylation level at the SLCO4C1 promoter (cg06480736, cg19774478, cg19788741 and cg22149516) (Table 2). Furthermore, multivariate Cox regression based on 4 different analyses, each including one CpG site, suggested that methylation levels at the SLCO4C1 promoter (cg06480736, cg19774478 and cg22149516) can be used as independent risk factors for BCR prediction (Table 3).

**Table 2:** Univariate Cox proportional hazards analysis of BCR-free survival in TCGA cohort including PC patients treated by radical prostatectomy

Variable	*P* value	Hazard ratio [95% CI]
Age (> 60 vs. ≤ 60)	0.8385	0.944 [0.545, 1.637]
pT3-4 vs. pT2	< 0.001	7.266 [2.617, 20.172]
pN1 vs. pN0	0.025	2.045 [1.094, 3.823]
Gleason score	< 0.001	4.244 [2.256, 7.981]
Preoperative PSA	0.0015	1.025 [1.009, 1.04]
R1 vs. R0	0.0231	1.902 [1.092, 3.312]
SLCO4C1 methylation		
cg06480736 (continuous)	< 0.001	15.914 [3.863, 65.565]
cg19774478 (continuous)	< 0.001	9.001 [2.789, 29.054]
cg19788741 (continuous)	0.003	10.759 [2.203, 52.553]
cg22149516 (continuous)	0.006	17.144 [2.295, 128.066]

**Table 3:** Multivariate Cox proportional hazards analysis of BCR-free survival in TCGA cohort including PC patients treated by radical prostatectomy

Variable	*P* value	Hazard ratio [95% CI]
cg19788741 (continuous)	0.087	3.927 [0.815, 18.904]
pT3-4 vs. pT2	0.031	3.843 [1.123, 13.154]
pN1 vs. pN0	0.968	0.987 [0.516, 1.886]
Gleason score	0.009	2.587 [1.267, 5.280]
Preoperative PSA	0.051	1.018 [0.999, 1.036]
cg06480736 (continuous)	0.049	1.809 [1.175, 13.769]
pT3-4 vs. pT2	0.026	4.022 [2.617, 20.172]
pN1 vs. pN0	0.993	1.002 [0.525, 1.941]
Gleason score	0.011	2.528 [1.234, 5.179]
Preoperative PSA	0.064	1.016 [0.999, 1.034]
cg19774478 (continuous)	0.041	1.903 [1.025, 3.534]
pT3-4 vs. pT2	0.045	3.532 [1.023, 12.199]
pN1 vs. pN0	0.826	0.092 [0.485, 1.780]
Gleason score	0.005	2.744 [1.344, 5.602]
Preoperative PSA	0.047	1.018 [1.000, 1.037]
cg22149516 (continuous)	0.008	2.316 [1.233, 4.350]
pT3-4 vs. pT2	0.040	3.662 [1.055, 12.710]
pN1 vs. pN0	0.972	1.011 [0.530, 1.927]
Gleason score	0.018	2.385 [1.155, 4.925]
Preoperative PSA	0.031	1.019 [1.001, 1.037]

## Discussion

Epigenetic modifications play an important role in the development of tumours. Epigenetic modifications do not normally alter the sequence of DNA. Once genes undergoes heritable changes through processes such as cell proliferation or differentiation, alternative splicing and genetic imprinting, DNA methylation can take place, representing the most common molecular mechanism of epigenetic modification [11–13]. Previous studies have shown that DNA promoter methylation is significantly related to the development of different tumours. In recent years, aberrantly methylated promotors have been explored as potential biomarkers for the diagnosis and prognosis of PC [14, 15]. The results published by Kirby et al., who generated the GSE76938 dataset, showed that DNA methylation patterns are altered in prostate cancer tissues in comparison to those in benign adjacent tissues and have a high predictive power for distinguishing malignant prostate tissues from benign-adjacent prostate tissues [16]. In this study, we focused on the differential methylation of CpG sites, which are located in the TSS200-1500 region of the gene. Then, a total of 10,206 qualified CpG sites were filtered from the GEO dataset GSE76938 using R software 3.1.4. To screen for high-quality differentially methylated CpG sites associated with gene functions, we focused on CpG sites with high levels of methylation, which were negatively correlated with gene expression. Finally, we identified 59 candidate genes. These novel candidate genes included several genes that have been known to be frequently hypermethylated in PC, such as TWIST1, ZNF154, S100A2, PTGS2, PON3, AOX1 and HIF3A. The AOX1 gene, contributing to improved diagnostic sensitivity and related to poor prognosis in PC patients, can be used as an independent risk factor for predicting cancer recurrence in PC [17, 18]. HIF3A is associated with the promotion of PC progression and can also be used as a biomarker for PC diagnosis [19]. PTGS2 has been associated with adverse clinical outcomes in several malignancies, including PC. High methylation of PTGS2 indicates a more than fourfold increased risk of BCR in PC [20].

Then, the top candidate gene SLCO4C1, which includes the four qualified sites (cg06480736, cg19774478, cg19788741 and cg22149516), was selected for further analysis. The results showed that SLCO4C1 harbours a relatively higher level of methylation in tumour tissues from a validation cohort (TCGA). Furthermore, BSAS confirmed a high level of methylation at the SLCO4C1 promoter in tumour tissues and a relatively low level of methylation in BPH tissues. Among these CpG sites on the SLCO4C1 promoter, differential methylation of the CpG sites cg06480736 and cg19788741 was the most significant in both the GSE76938 dataset and TCGA database. Based on analysis of data from TCGA, the expression of SLCO4C1 was negatively correlated with the levels of methylation at these four CpG sites (cg06480736, cg19774478, cg19788741 and cg22149516). Two sites (cg19788741 and cg22149516) were significantly associated with a low expression of SLCO4C1. qRT-PCR results further revealed that SLCO4C1 was significantly downregulated in PC samples. Based on the above analysis, we infer that aberrant methylation of the SLCO4C1 promoter, especially at the site cg19788741, may be correlated with genetic events in PC, contributing to SLCO4C1 expression and function, cancer initiation, progression, invasion and recurrence. However, a few studies have reported SLCO4C1 as a potential tumour suppressor gene and shown that low expression of SLCO4C1 may be associated with the development of tumours [21]. The function and molecular mechanisms of SLCO4C1 in PC are not clear thus far. High-quality molecular biology experiments are needed to confirm the above conclusions.

In the analysis of clinicopathological parameters, the results showed that methylation levels at all four sites on the SLCO4C1 gene were related to advanced tumour stages. In contrast, preoperative PSA values were not associated with high methylation levels at any of the four sites on the SLCO4C1 gene. Methylation levels at sites cg06480736, cg19774478 and cg19788741 of SLCO4C1 were related to Gleason score, surgical margin status and nodal category. Although the statistical results showed that methylation levels at two sites (cg06480736 and cg19788741) are related to age, this result may be due to the statistical methods used and small sample sizes. In summary, our evidence supports the idea that hypermethylation in at least three sites inhibit the expression of SLCO4C1, which means that SLCO4C1 promoter methylation may play an important role in PC progression and that SLCO4C1 could be used potentially as a therapeutic target. In this study, there were no experiments to verify the gene functional changes after methylation. Finally, many other experiments are needed to verify this conclusion.

## Conclusions

All four CpG sites (cg06480736, cg19774478, cg19788741 and cg22149516) on the SLCO4C1 promotor were significantly associated with BCR, and high methylation levels at the SLCO4C1 promotor predicted poor outcomes for PC by Kaplan-Meier analysis. Hypermethylation of SLCO4C1 (cg06480736, cg19774478 and cg22149516) indicates a higher chance of BCR based on multivariate Cox regression analysis. This evidence supports the notion that hypermethylation of SLCO4C1 leads to gene expression inhibition and functional loss, suggesting that SLCO4C1 could be used as a potential biomarker for prognosis associated with biochemical recurrence-free survival after radical prostatectomy. This marker might be helpful for the distinction between aggressive and indolent prostate cancer.

## Materials and methods

### Database and data analysis

The GSE76938 dataset was downloaded from the Gene Expression Omnibus (GEO) database (https://www.ncbi.nlm.nih.gov/geo/). This dataset includes 73 cases of PC and 63 cases of adjacent tissue. Total genome methylation data were collected until 2017. The validation cohort was based on data collected from The Cancer Genome Atlas (TCGA), downloaded from the database (https://www.cancer.gov/). This dataset comprises data from 498 patients with histologically confirmed PC obtained from international centres. SLCO4C1 mRNA expression data are also available in these two databases. Biochemical recurrence-free survival (BCR-free survival) was considered the primary endpoint of the study. The format of data downloaded from TCGA data was level 3. DNA methylation profiles were analysed using the Illumina Infinium Human Methylation 450 assay platform, and the DNA beta value was converted to *M*-values. For the convenience of Statistics by R 3.1.4 minfi package, the normalized *M*-values instead of *M*-values were processed by *Z*-Score method. A higher value represented a higher level of methylation. RNA sequencing profiles were analysed using the Illumina HiSeq 2000 RNA experimental platform.

### Screening for methylated genes

To identify highly cancer-specific CpG sites in GSE76938, we employed a strict threshold in which only genes with |Δ*β*| > 0.2 and *P* < 0.001 were included in this study. These CpG sites were evaluated by the limma package for linear regression model analysis with patients as a random effect, and degree of methylation (|Δ*β*|), status of biochemical recurrence, age and ethnicity as variables.

After removing the differentially methylated CpG sites that were not located in the TSS200 region of the gene promoter, a total of 10,206 qualified CpG sites were found in GSE76938. Using these CpG sites and gene annotation files, we found 2182 genes that displayed promoter methylation. We selected the top significant differential gene SLCO4C1 for further study. After further analysis, four differentially methylated CpG sites (cg06480736, cg19774478, cg19788741 and cg22149516) were found to be located in the TSS200 region of the SLCO4C1 promotor. Although these four sites were all negatively correlated with SLCO4C1 expression, the sites cg19788741 and cg06480736 produced the most significant results.

### Co-expression and functional enrichment analyses

SLCO4C1 RNA expression in PC and normal prostate tissues was obtained from Oncomine (www.oncomine.org). The top 100 co-expressed genes were extracted from the GEPIA database (http://gepia.cancerpku.cn/index.html). TCGA PRAD tumour and TCGA PRAD normal datasets were used to perform co-expression analyses. Then, these co-expressed genes were included in WebGestalt (http://www.webgestalt.org/option.php) to perform gene ontology analysis.

### Sample preparation

A total of 20 PC and benign prostatic hyperplasia (BPH) tissue samples were acquired from The First Affiliated Hospital of Chongqing Medical University. All patients had been diagnosed by pathological assessment of tissue biopsy and had undergone surgeries between October 2016 and July 2018. No radiotherapy or chemotherapy was performed before the samples were collected. The study was approved by the Research Ethics Committee of Chongqing Medical University. Informed consent was obtained from all participating patients.

### Bisulfite amplicon sequencing (BSAS) and total RNA extraction

Bisulfite amplicon sequencing (BSAS) was performed within CpG sites of the SLCO4C1 in the same region as the four sites selected from TCGA. The primers specific for SLCO4C1 were forward 5′-TAAGGGGAGTTTATGGTTAGAGAT-3′ and reverse 5′-AAAAARGAAAATTCTCACCCC-3′. Then, MethylKIT software (http://www.bioconductor.org/packages/release/bioc/html/methylKit.html) was used to analyse methylation data and calculate the average methylation level at all sites. Total RNA was extracted using TRIzol reagent (TaKaRa). cDNA reverse transcription and qRT-PCR were performed following the instructions of Reverse Transcription PrimeScript 1st Stand cDNA Synthesis kit (TaKaRa) and quantitative PCR reagents SYBR Premix ExTaq™ (TaKaRa). β-Actin was used as the corresponding internal control. All mRNA levels were quantified by the 2^−ΔΔCT^ method. Each reaction was performed in triplicate. The primers specific for SLCO4C1 were forward 5′-AGGGGCCATCCACAGTCTTC-3′ and reverse 5′-AAGACCCCTCCTCAAACTCG-3′.

### Statistical analysis

R software version 3.1.4 was used to assess the relationship between the four hypermethylation sites and mRNA expression by Spearman’s correlation analysis. Kaplan-Meier analysis and univariate and multivariate Cox regression were used to assess whether the four methylation sites were associated with BCR. The Mann-Whitney *U* test and Kruskal-Wallis test were used to analyse the correlation between SLCO4C1 and clinicopathological parameters. In the multivariate Cox regression, 4 different analyses were performed, each including one CpG site.

## Data Availability

Not applicable.

## Abbreviations

BCR: Biochemical recurrence TCGA The Cancer Genome Atlas
BSAS: Bisulfite amplicon sequencing
CRPC: Castration-resistant prostate cancer
GEO: Gene Expression Omnibus
PC: Prostate cancer
PSA: Prostate-specific antigen
qRT-PCR: Quantitative real-time polymerase chain reaction
